# REVERSE-NAMASKAR: A NEW SIGN IN EHLERS-DANLOS SYNDROME: A FAMILY PEDIGREE STUDY OF FOUR GENERATIONS

**DOI:** 10.4103/0019-5154.60360

**Published:** 2010

**Authors:** S Premalatha, K N Sarveswari, Koushik Lahiri

**Affiliations:** *From Former Department of Dermatology, Stanley Medical College and Hospital, Chennai, India.*; 1 *From Former Sundaram Medical Foundation, Dr. Rangarajan Memorial Hospital, Chennai, India.*; 3 *From Former Apollo Gleneagles Hospitals, Kolkata, India.*

**Keywords:** *Ehlers-Danlos syndrome*, *‘Reverse-Namaskar’ sign*, *signs in Ehlers-Danlos syndrome*

## Abstract

Ehlers-Danlos Syndrome (EDS) is a rare group of inheritable connective tissue disorder of defective collagen. Skin, joints and blood vessels are most commonly affected. Clinical signs such as Gorlin sign and Metenier sign have been described in this syndrome. We report another new clinical sign called ‘Reverse-Namaskar’ sign as an important clinical finding in EDS, based on the family pedigree study of the proband.

## Introduction

Ehlers-Danlos syndrome (EDS) belongs to a heterogeneous group of disorders of collagen characterized by hyper extensibility and fragility of the skin with easy bruisability and hyper mobility of the joints.[1] It is a rare genetic disorder affecting 1 in 5000 people.[2]

The diagnosis in classic EDS (EDS I and II) is established mainly by the family history and the characteristic clinical findings.

EDS is caused by underlying ultra structural abnormalities in the collagen fibrils, which is genetically determined.[3] Cutaneous, vascular, skeletal, ocular and dental manifestations have been described.[4] The phenotype varies depending upon the type of collagen altered.

In a recent consensus in Villefranche, in 1997, the classification of EDS was reorganized into six major subtypes.[3] The diagnostic skin signs described in EDS includes Gorlin sign (ability to touch the tip of the nose with the tongue), Metenier sign (easy eversion of upper eyelid) and atrophic ‘cigarette paper’ scarring.[5,6] Molluscoid pseudo tumors and spheroids may occur in EDS.[3,5] These are subcutaneous nodules due to herniation of subcutaneous fatty tissue and resemble lipomas histologically.

We came across two family members of this pedigree in four generations showing a new sign, which we would like to label as ‘Reverse-Namaskar’ sign.

## Case Report

**Figure 1:**
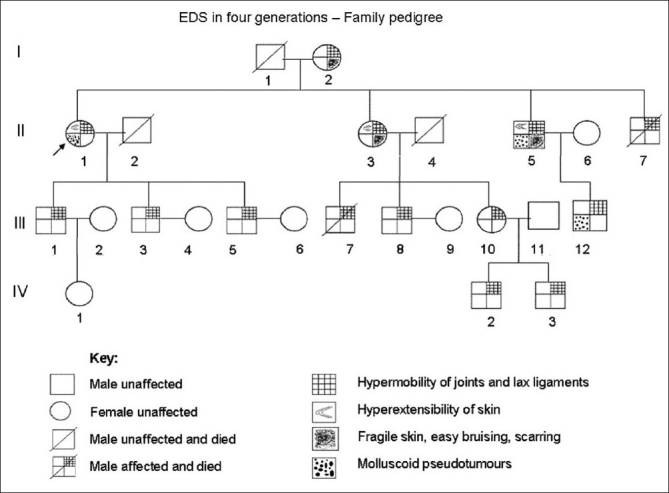
Family pedigree chart - Ehlers-Danlos syndrome in four generations

Clinical details are shown in the family pedigree chart [Figure 1].

### II-1. Proband

A 68-year-old female scientist presented with asymptomatic multiple firm subcutaneous nodular lesions over both the upper extremities since two decades. She gave history of frequent falls since childhood, either due to sudden inversion of left foot or sudden flexion of left knee.

She had also suffered from sprain of her left foot on many occasions and a fracture of 5^th^ metratarsal bone at 23 years of age. Her first child was born at eight months due to premature rupture of the amniotic membrane.

There was history of frequent subluxation and mild dislocation of her right shoulder joint associated with occasional pain since 20 years. She had an accident due to sudden inversion of her left foot with subsequent fracture of both tibia and fibula with crushed left medial malleolus, which was surgically corrected. Apart from a meniscal tear of her left knee joint a few months back, she also had a recent fall with fracture of middle phalanx of left 3^rd^ toe.

She was highly myopic since childhood and recently underwent cataract surgery in both eyes, corrected with IOL. She was neither a diabetic nor a hypertensive.

### On Examination

She was of average height and slightly obese. General and systemic examinations revealed no abnormality. No hyper mobility of the joints was seen. Mild hyper extensible skin was present over her forearms and dorsa of the hands.

**Figure 2:**
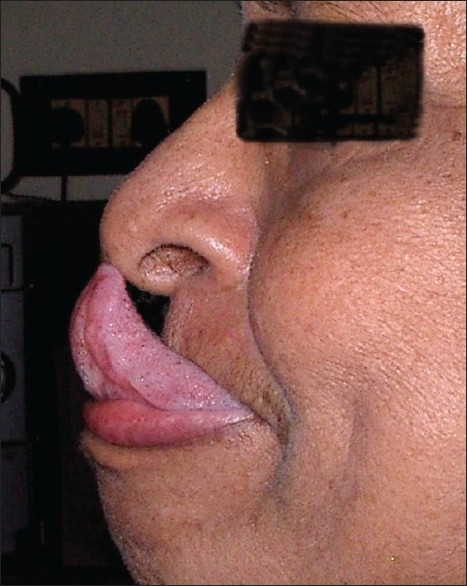
Gorlin sign in proband

Gorlin sign was positive [Figure 2]. Multiple painless and non-tender firm mobile subcutaneous nodules of various sizes ranging from 1 cm to 2 cm were present over her upper extremities, mostly over the lateral aspect of her right arm and flexor aspect of her right forearm. These were only palpable nodules and not visible lesions, suggestive of spheroids. Telangiectasia and superficial veins were seen through the apparently thin translucent skin above both knees. She had an asymptomatic piezogenic pedal papule on her right heel.

**Figure 3:**
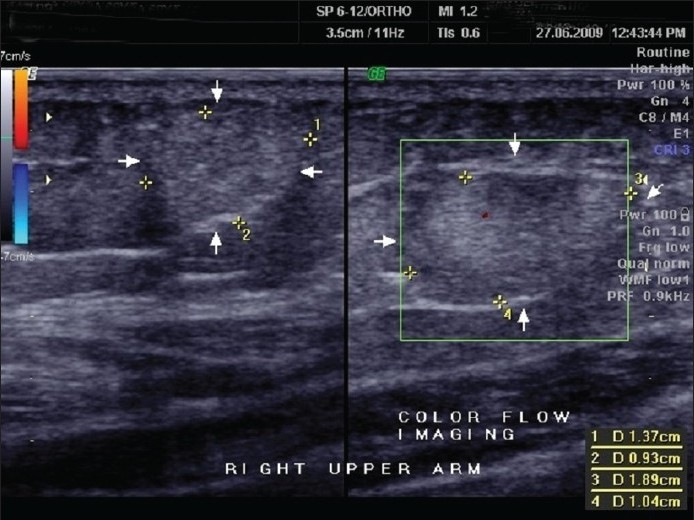
USG of spheroids right arm in proband in favor of circumscribed encapsulated tumor fibrolipoma

Routine blood and urine examination were normal. GTT revealed impaired glucose tolerance. Lipid profile was normal. Skiagram of the chest was normal. USG of the right arm and forearm showed subcutaneous encapsulated tumors in favour of lipomas [Figure 3]. Skin biopsy was done from the hyperextensible skin over the left forearm.

**Figure 4:**
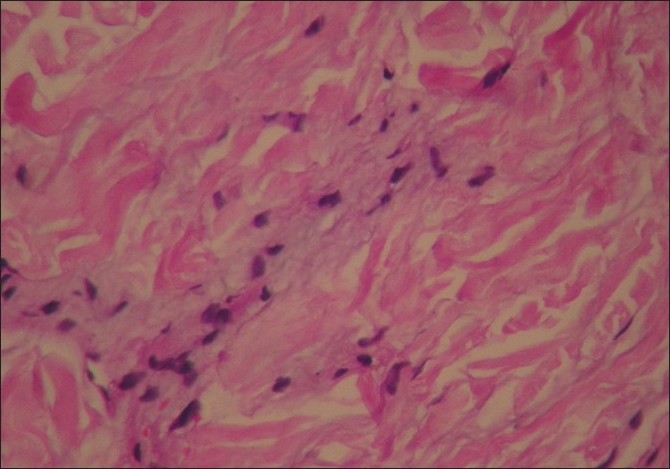
H and E (high magnification) shows myxoid degeneration of collagen in the dermis

**Figure 5:**
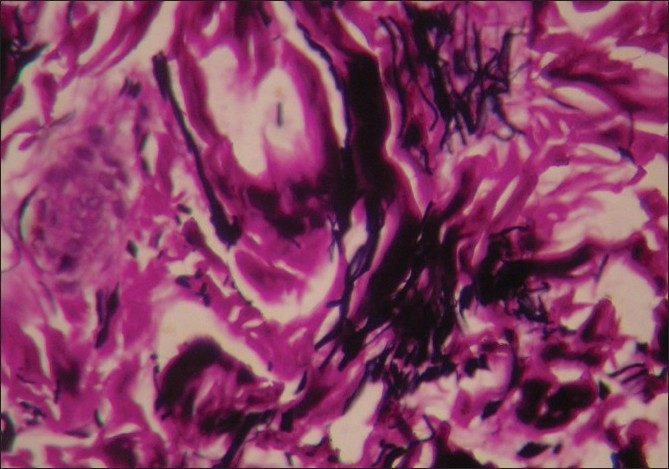
Elastic tissue stain Verhoeff-Van Gieson (high magnification) shows relative increase of elastic fibers in the dermis

Routine haematoxyline and eosin stain revealed flattening of the epidermal rete ridges and mild myxoid degeneration of collagen in the dermis.[Figure 4] Elastic tissue stain showed a relative increase of elastic fibers in some areas of the dermis.[Figure 5]. Histopathological features were in favor of EDS light microscopically.

### I-2

An 88-year-old female, mother of the proband, gave history of subluxation of her right shoulder joint on and off since 30 years. Her skin was of soft texture with easy bruisability on trauma.

### II-3

A 65-year-old homemaker and sister of the proband gave history of frequent falls and dislocation/subluxation of shoulder joints now and then for the past 30 years.

## On Examination

**Figure 6:**
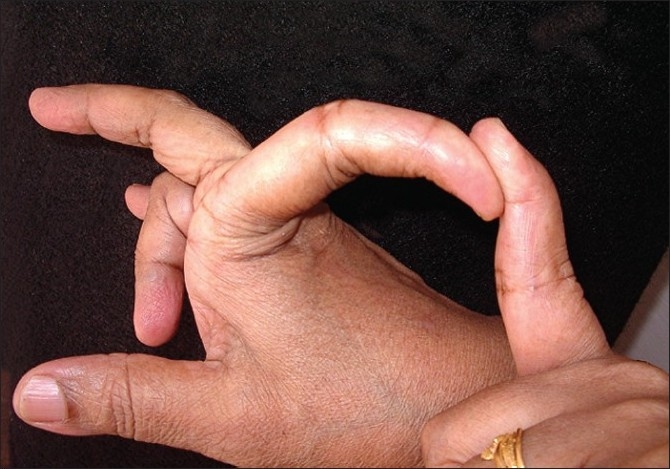
Hyper extensibility of the fingers in proband's sister

**Figure 7:**
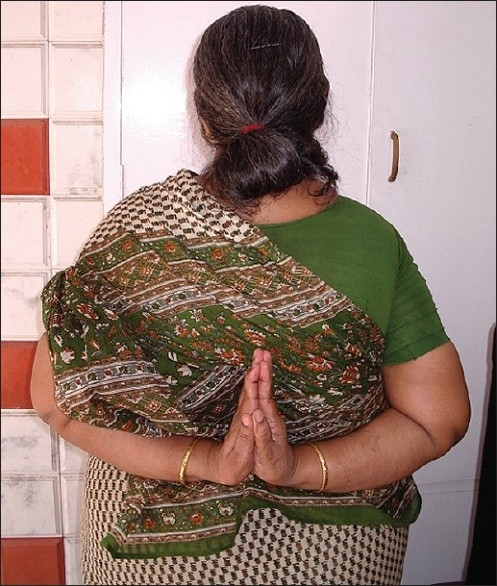
‘Reverse-Namaskar’ sign due to lax joints in proband's sister

**Figure 8:**
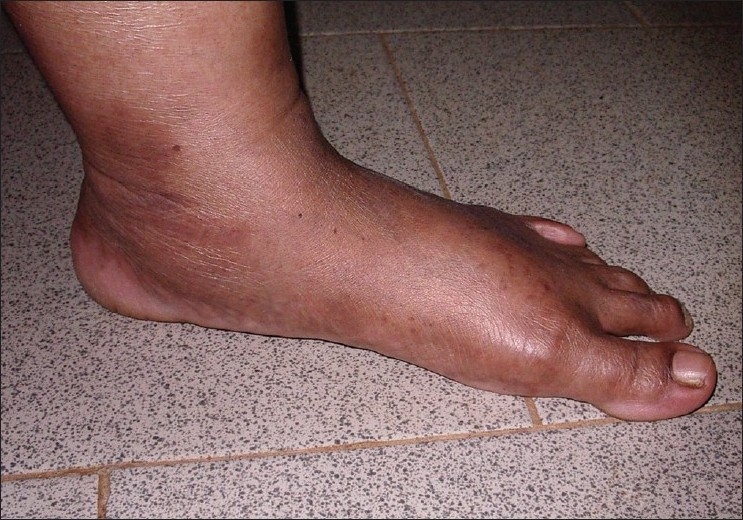
Flat foot in proband's sister

She was of average height and obese. She had lax joints with hyper extensibility of all fingers [Figure 6]. ‘Reverse - Namaskar’ sign was present [Figure 7]. She was able to fold her forearms at the back and to oppose her palms facing each other to say ‘Namaskar’. Her Skin was soft with easy bruising. Varicose veins were present over her lower extremities. She had pes planus of both feet [Figure 8].

### II-5

A 60-year-old male, proband's brother, gave history of easy bruising of the skin since childhood. There was history of painful dislocation of left shoulder joint on and off since three decades. This was surgically corrected 10 years back. He had frequent subluxation/dislocation of right hip joint in the past few years. Inguinal hernia (left side) was operated at 15 years of age. He also gave history of reflux esophagitis since eightyears.

## On Examination

**Figure 9:**
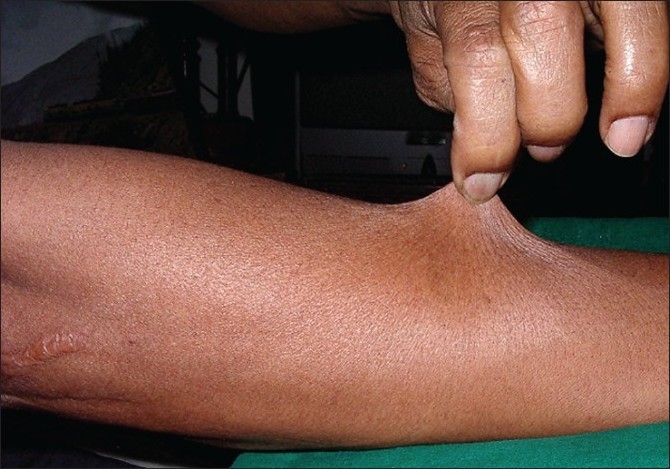
Hyper extensibility of the skin with tissue paper like scar near the elbow-proband's brother

**Figure 10:**
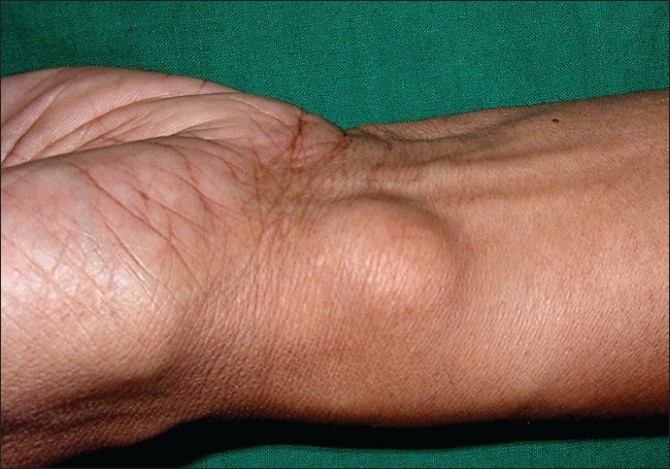
Molluscoid pseudo tumor over the wrist of proband's brother

Hyper extensible skin was present over the forearms [Figure 9] with ‘cigarette paper’ like scars due to trauma. Asymptomatic multiple subcutaneous small firm mobile nodules were palpable over both arms. A few firm nodules were visible over the abdomen and left forearm suggestive of molluscoid pseudo tumors [Figure 10].

### II-7

Another brother of proband with marfanoid features had hyper mobility of right shoulder joint, died two years back, at the age of 56, due to esophageal carcinoma with metastasis in the liver.

### III-1

A 42-year-old male, first son of the proband, had laxity of the interphalangeal joint of the left thumb, since childhood. No other manifestation of EDS was present.

### III-3

A 37-year-old male, second son of the proband gave history of. subluxation of both shoulder joints now and then for the past 15 years. He has been highly myopic since childhood. He had hyper extensibility of all fingers and mild hyper extensible skin.

### III-5

A 35-year-old male, third son of the proband, gave history of subluxation of both shoulder joints and right hip joint on and off since 10 years. Left elbow joint was hyper extensible. Four supernumerary teeth in the maxilla and one supernumerary tooth in the mandible had been removed. Gorlin sign and Metenier sign were present.

### III-7 and III-8

Both the sons of II-3 had hyper mobility of shoulder joints with history of dislocation now and then. The eldest son (III-7) died at the age of 25 years due to an accident.

### III-10

A 35-year-old homemaker, the daughter of II-3, had hyper mobility of right thumb since childhood. No other clinical manifestations of EDS at present.

### III-12

A 23-year-old male, son of II-5, had laxity of both shoulder and knee joints with subluxation of both shoulder joints on and off since 10 years and lax proximal metacarpo phalangial joint of left thumb since childhood. There was no history of easy bruising or hyper extensibility of skin.

**Figure 11:**
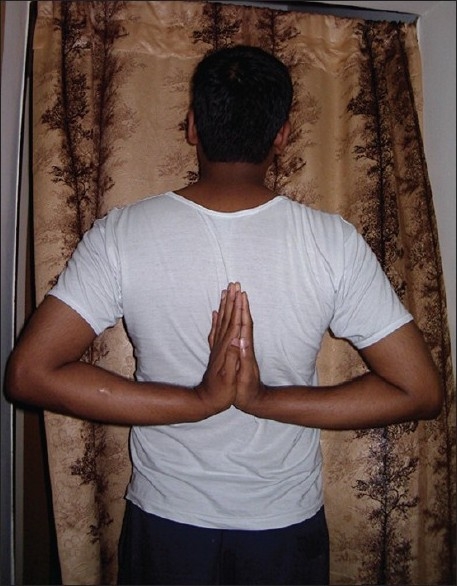
‘Reverse-Namaskar' sign, in proband's brother's son

‘Reverse-Namaskar’ sign of the authors was present [Figure 11]. There were multiple firm visible non-tender subcutaneous nodules over the upper extremities and back. Two large nodules were excised about five years back for cosmetic reasons and histopathology was in favor of molluscoid pseudo tumors. He was highly myopic in childhood, which was corrected with spectacles.

### IV-2 and IV-3

The two children of III-10, aged 12 and 7, presented with hyper extensible thumbs. They showed no other features of EDS. IV-2 had myopia corrected with glasses.

**Figure 12:**
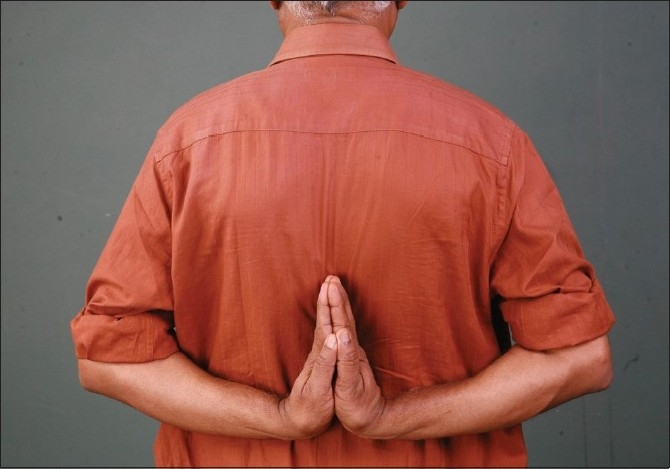
‘Reverse-Namaskar’ sign in a relative of the second author

**Figure 13:**
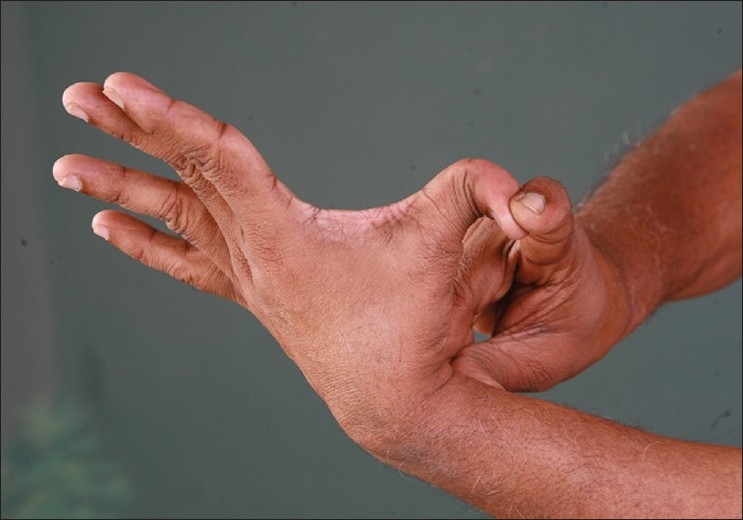
Hyper extensibility of thumb in the same relative of the second author

Interestingly, the following were noted by coincidence:

One of the relatives of the second author demonstrated the ‘Reverse-Namaskar’ sign [Figure 12]. He also had hyper extensibility of both thumbs [Figure 13]. His daughter also demonstrated the ‘Reverse-Namaskar’ and ‘Gorlin’ signs.

**Figure 14:**
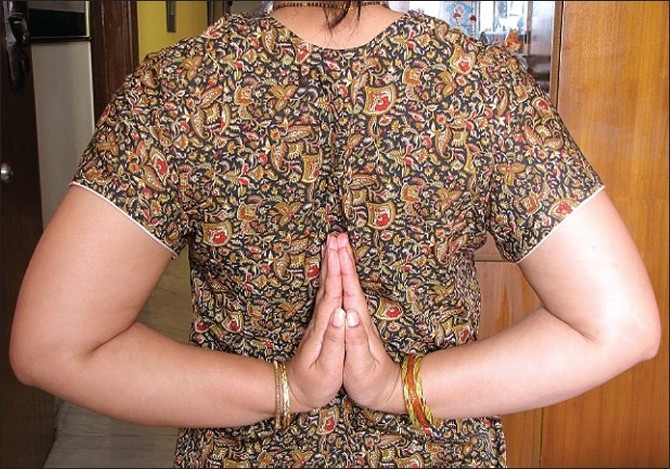
'Reverse- Namaskar' sign in a relative of the third author

One of the relatives of the third author also showed the ‘Reverse-Namaskar’ sign [Figure 14]. She had no other manifestations of EDS. Her daughter also revealed this sign without any other manifestations of EDS.

## Discussion

To our knowledge, the ‘Reverse-Namaskar’ sign, an interesting sign in EDS is hitherto unreported in literature. ‘Namaskar’ is the typical Indian way of greeting people, where the forearms are folded in front of the chest and the palms are opposed together. Here, two family members of the proband were able to fold their forearms at the back and oppose their palms to say ‘Namaskar’ demonstrating the hyper extensible joints. This sign, used by the affected individuals to amuse their friends during their childhood days, could well be a valuable diagnostic clue to EDS.

In the present study hyper extensible skin with easy bruisability was seen in II-1, II-3 and II-5. Molluscoid pseudo tumors and spheroids were present in the proband, II-5 and III-12. Hyper mobility of the joints is commonly seen in EDS I, II, III and VII of dominant inheritance, and EDS VI of recessive inheritance.[3] Hyper mobility with subluxation of the joints was the common finding in all family members in this study. Dislocation of shoulder joint was seen in II-5.

Ocular manifestations like myopia, Metenier sign and strabismus are present in EDS type I, VI and VII.[6] In the present study myopia was seen in the proband, III-3 and IV-2. Metenier sign was demonstrated in III-5. He also had supernumerary teeth, very rarely reported in literature.[4,7]

In view of the major skin and joint manifestations present in four generations of this family pedigree study, the authors consider that the cases fit in with Classic (I and II) types with overlapping of type III of EDS of dominant inheritance. ‘Familial joint hyper mobility syndrome’, a close differential diagnosis has been excluded because of the presence of the characteristic skin changes of EDS associated with laxity and hyper mobility of the joints seen in our study. The relative increase in elastic fibers with mild degenerative changes in collagen proved by special stain in our case is consistent with the literature finding.[8]

## Conclusion

This new ‘Reverse-Namaskar’ sign, if present along with other characteristic features of EDS, may be considered a valuable diagnostic sign of EDS indicating hyper extensibility of the joints. This sign has to be looked for in cases of ‘familial joint hyper mobility syndrome’ also in future for its significance.

Secondly, the authors feel that EDS may not belong to ‘rare genodermatosis’. The prevalence of EDS in literature varies between 1/10,000 and 1/25,000.[3]

EDS is just ‘uncommon’. Many people may have EDS as forme fruste, having minor collagen deficit, carrying on their routine normal life.

EDS is often unnoticed or the diagnosis is missed frequently, especially in cases of dominant inheritance. Only people with severe manifestations approach the doctor for opinion and treatment. The reported statistical incidence of EDS cases in literature may thus be low.

Thirdly, ‘Reverse-Namaskar,’ an important clinical sign has to be looked for in all cases of EDS in future, for its significance and its incidence in normal individuals also as controls, which may be similar to that of Gorlin sign presents in 50% of EDS and 10% of normal individuals.[5]

Finally, cases of EDS with this ‘Reverse-Namaskar’ sign have to be studied in detail in future with ultrastuctural study of the collagen fibrils and molecular genetic tests to evaluate the exact etiopathogenesis.
